# Supplementary material for: Entropic singularities give rise to quantum transmission
Source: Nat Commun. 2021 Oct 1;12:5750. doi: 10.1038/s41467-021-25954-0 (PMC8486852; doi:10.1038/s41467-021-25954-0)
Supplement: Supplementary file 1 — Supplementary Information [file 41467_2021_25954_MOESM1_ESM.pdf]

Supplementary Information: Entropic singularities give rise to quantum transmission

Vikesh Siddhu[1,2*]

Date: August 31, 2021

Supplementary Note 1: Preliminaries

Let $\mathcal{H}$ denote a d-dimensional Hilbert space and $\hat{\mathcal{H}}$ the space of linear operators on $\mathcal{H}$. The l_1 norm of any operator $A \in \hat{\mathcal{H}}$, $|A|_1 := \mathrm{Tr}(\sqrt{AA^\dagger})$, defines an l_1 distance between two such operators A and B, $l_1(A,B) = |A-B|_1$. Consider an isometry,

$$J : \mathcal{H}_a \mapsto \mathcal{H}_b \otimes \mathcal{H}_c; \quad J^\dagger J = I_a \tag{1}$$

that maps an input Hilbert space $\mathcal{H}_a$ (of dimension d_a) to a subspace of a pair of output spaces $\mathcal{H}_b \otimes \mathcal{H}_c$. In the main text, we have used a more concise notation a, and $b \otimes c$ to refer to $\mathcal{H}_a$ and $\mathcal{H}_b \otimes \mathcal{H}_c$, respectively. For completeness, we repeat some material from the main text. The isometry J generates a noisy quantum channel pair $(\mathcal{B}, \mathcal{C})$ with superoperators,

$$\mathcal{B}(A) = \mathrm{Tr}_c(JAJ^\dagger), \quad \text{and} \quad \mathcal{C}(A) = \mathrm{Tr}_b(JAJ^\dagger), \tag{2}$$

that take any element A of $\hat{\mathcal{H}}_a$ to $\hat{\mathcal{H}}_b$ and $\hat{\mathcal{H}}_c$, respectively. Both channels in this $(\mathcal{B}, \mathcal{C})$ pair represent a completely positive trace preserving map, and each channel may be referred to as the complement of the other channel. The dimensions d_b and d_c, of outputs $\mathcal{H}_b$ and $\mathcal{H}_c$, respectively are the ranks of $\mathcal{B}(I_a)$ and $\mathcal{C}(I_a)$, respectively. These are the smallest possible output dimensions required to define $(\mathcal{B}, \mathcal{C})$ (in the notation of Def. 4.4.4 in [1], d_b is the Choi-rank of $\mathcal{C}$ and d_c is the Choi-rank of $\mathcal{B}$). These definitions make the channel pair setting symmetric with respect to replacement of one channel in the pair with its complement. In the main text, we sometimes write $\mathcal{B} : a \mapsto b$, to represent that the quantum channel $\mathcal{B}$ takes operators on $\mathcal{H}_a$ to operators on $\mathcal{H}_b$.

Let ρ be a density operator with eigenvalues $\{\lambda_i\}$ then,

$$S(\rho) = -\mathrm{Tr}(\rho \log \rho) = -\sum_i \lambda_i \log \lambda_i, \tag{3}$$

denotes the von-Neumann entropy of ρ. For any input density operator ρ_a in $\hat{\mathcal{H}}_a$, ρ_b and ρ_c denote the outputs $\mathcal{B}(\rho_a)$ and $\mathcal{C}(\rho_a)$, respectively. The *entropy bias* or the *coherent information* of $\mathcal{B}$ at ρ_a is

$$\Delta(\mathcal{B}, \rho_a) = S(\rho_b) - S(\rho_c). \tag{4}$$

The *channel coherent information*,

$$\mathcal{Q}^{(1)}(\mathcal{B}) = \max_{\rho_a} \Delta(\mathcal{B}, \rho_a). \tag{5}$$

If there is a channel $\mathcal{D}$ such that $\mathcal{C} = \mathcal{D} \circ \mathcal{B}$, then $\mathcal{B}$ is said to be *degradable* and $\mathcal{Q}(\mathcal{B}) = \mathcal{Q}^{(1)}(\mathcal{B})$, $\mathcal{C}$ is said to be *antidegradable* and $\mathcal{Q}^{(1)}(\mathcal{C}) = \mathcal{Q}(\mathcal{C}) = 0$ [2].

In what follow, we use the notation $[\psi]$ for the dyad $|\psi\rangle\langle\psi|$. First, in Supplementary Note (SN) 2, we discuss the relationship between log-singularities and continuity bounds. Next, in SN-3, we discuss properties of channel outputs at rank 1 and full rank inputs. In SN-4, we discuss some technical details about applications of Theorem 1 in the main text, where SN-4.3 uses some results from SN-3. Finally, SN-4 has mathematical details about the isometry, J_1, and the channel coherent information, $\mathcal{Q}^{(1)}(\mathcal{B}_1)$, of the low-noise channel, $\mathcal{B}_1$, all introduced in the main text.

[1] Department of Physics, Carnegie Mellon University, Pittsburgh, Pennsylvania 15213, U.S.A, [2] JILA, University of Colorado/NIST, Boulder, CO 80309, U.S.A (current address); *email: vsiddhu@protonmail.com

Supplementary Note 2: Continuity and log-singularity

An $\epsilon \log$-singularity in the von-Neumann entropy can dominate continuity bounds on this entropy. A simple continuity bound on the von-Neumann entropy comes from the Fannes–Audenaert inequality [3,4]. If the l_1 distance between two d-dimensional density operators ρ and σ is 2ϵ, that is, $|\rho - \sigma|_1 = 2\epsilon$, then

$$|S(\rho) - S(\sigma)| \leq \epsilon \log(d-1) + h(\epsilon), \tag{6}$$

where $h(\epsilon) := -[\epsilon \log_2 \epsilon + (1-\epsilon)\log_2(1-\epsilon)]$ is the binary entropy function. Suppose $\rho(\epsilon) = (1-\epsilon)[0] + \epsilon[1]$ is a qubit density operator where $0 \leq \epsilon \leq 1$, and $\sigma = \rho(0)$. Then $\rho(\epsilon)$ and σ have l_1 distance 2ϵ and satisfy (6). Notice, in the present case, the von-Neumann entropy of $\rho(\epsilon)$ has an $\epsilon \log$-singularity and the right side of the inequality (6) is simply $h(\epsilon)$. The binary entropy function $h(\epsilon)$ has an $\epsilon \log$-singularity in the sense that for small ϵ, $dh(\epsilon)/d\epsilon \simeq O\big(\log(\epsilon)\big)$; that is, the gradient of the upper bound (6) is logarithmic in ϵ and tends to infinity as ϵ tends to zero. In this sense, for small ϵ, the continuity upper bound is essentially dominated by a logarithmic singularity.

A continuity upper bound on the coherent information comes from the Alick-Fannes-Winter (AFW) inequality [5,6]. We find that log-singularities can also dominate the behavior of this bound. A bipartite density operator ρ_{ab} on $\mathcal{H}_{ab} := \mathcal{H}_a \otimes \mathcal{H}_b$ has coherent information $I_{a>b}(\rho_{ab}) := S(\rho_b) - S(\rho_{ab})$. The AFW inequality shows that if the l_1 distance between two density operators ρ_{ab} and σ_{ab} is at most 2ϵ, $|\rho_{ab} - \sigma_{ab}|_1 \leq 2\epsilon$, then coherent information of ρ_{ab} and σ_{ab} satisfy an inequality,

$$|I_{a>b}(\rho_{ab}) - I_{a>b}(\sigma_{ab})| \leq 2\epsilon \log d_a + (1+\epsilon) h\big(\frac{\epsilon}{1+\epsilon}\big). \tag{7}$$

Consider a density operator,

$$\rho_{ab}(\epsilon) = (1-\epsilon)[00] + \epsilon[\phi], \tag{8}$$

where $|\phi\rangle_{ab} = \frac{1}{\sqrt{2}}(|01\rangle + |10\rangle)$, $|ij\rangle$ denotes $|i\rangle_a \otimes |j\rangle_b \in \mathcal{H}_a \otimes \mathcal{H}_b$, and let $\sigma_{ab} = \rho_{ab}(0)$. Then $\rho_{ab}(\epsilon)$ and σ_{ab} have l_1 distance 2ϵ and satisfy the AFW inequality (7). The von-Neumann entropy of $\rho_{ab}(\epsilon)$ has an $\epsilon \log$-singularity and for small ϵ, changes in $S\big(\rho_{ab}(\epsilon)\big)$ are logarithmic in ϵ. The upper bound in (7) also experiences similar logarithmic changes, that is, for small ϵ the gradient of the upper bound is $O(\log \epsilon)$ and this gradient tends to infinity as ϵ tends to zero.

Supplementary Note 3: Spectrum of channel outputs

3.1 Rank one inputs

Suppose an input ρ_a to a channel pair $(\mathcal{B}, \mathcal{C})$ (2) is a normalized pure state $[\psi]_a$, then the channel outputs, ρ_b and ρ_c, have the same spectrum, rank, and entropy. This elementary result is used at various places in the main text and this supplementary information write-up. To prove this result, we use the fact that two density operators with the same spectrum have equal rank and entropy and show that ρ_b and ρ_c have the same spectrum when $\rho_a = [\psi]_a$. To obtain this spectrum consider the action of J on the normalized ket $|\psi\rangle_a$,

$$J|\psi\rangle_a = |\psi\rangle_{bc} = \sum_i q_i |\beta_i\rangle_b \otimes |\gamma_i\rangle_c, \tag{9}$$

where $q_i > 0$ are Schmidt coefficients with $\sum_i q_i^2 = 1$, and $\{|\beta_i\rangle_b\}$ and $\{|\gamma_i\rangle_c\}$ are orthonormal kets in $\mathcal{H}_b$ and $\mathcal{H}_c$, respectively. Using the Schmidt decomposition (9) and eq. (2) we obtain,

$$\rho_b = \mathrm{Tr}_c([\psi]_{bc}) = \sum_i q_i^2 [\beta_i]_b, \quad \text{and} \quad \rho_c = \mathrm{Tr}_b([\psi]_{bc}) = \sum_i q_i^2 [\gamma_i]_c, \tag{10}$$

the spectral decompositions of ρ_b and ρ_c, respectively. These decompositions indicate that both channel outputs ρ_b and ρ_c have the same spectrum with eigenvalues $\{q_i^2\}$, thus proving our elementary result.

3.2 Full rank inputs

If a channel input ρ_a has rank d_a, then the channel outputs, ρ_b and ρ_c, have ranks d_b and d_c, respectively. To prove this statement, consider the spectral decomposition,

$$\rho_a = \sum_{i=1}^{d_a} p_i [\alpha_i]_a, \tag{11}$$

of a rank d_a density operator ρ_a, i.e., $\{p_i\}$ are d_a strictly positive eigenvalues that sum to unity and $\{|\alpha_i\rangle_a\}$ is an orthonormal basis of $\mathcal{H}_a$. Using this decomposition, ρ_b can be written as a convex combination of density operators $\mathcal{B}([\alpha_i]_a)$,

$$\rho_b = \sum_{i=1}^{d_a} p_i \mathcal{B}([\alpha_i]_a). \tag{12}$$

For any ket $|\phi\rangle_b \in \mathcal{H}_b$,

$$\mathrm{Tr}\big(\rho_b[\phi]_b\big) = \sum_i p_i \mathrm{Tr}\big(\mathcal{B}([\alpha_i]_a)[\phi]_b\big), \tag{13}$$

is a convex sum of non-negative numbers $\mathrm{Tr}\big(\mathcal{B}([\alpha_i]_a)[\phi]_b\big)$. This convex sum is strictly positive if each p_i is $1/d_a$ i.e., ρ_a in (11) is I_a/d_a and ρ_b in (12) is $\mathcal{B}(I_a)/d_a$, a rank d_b operator (see discussion below eq. (2)). This strict positivity of $\mathrm{Tr}\big(\rho_b[\phi]_b\big)$ at $p_i = 1/d_a$ implies for arbitrary $|\phi\rangle_b$,

$$\mathrm{Tr}\big(\mathcal{B}([\alpha_i]_a)[\phi]_b\big) > 0, \tag{14}$$

for some $|\alpha_i\rangle_a$. Notice $\mathrm{Tr}\big(\rho_b[\phi]_b\big)$ in (13) is the sum of non-negative numbers. The equation above implies that at least one of these numbers is strictly positive. Consequently, (13) is strictly positive for arbitrary $|\phi\rangle_b$. This strict positivity implies ρ_b is positive definite, i.e., ρ_b has rank d_b. A straightforward modification of the above reasoning shows that ρ_c has rank d_c when ρ_a is rank d_a.

4 Supplementary Note 4: Theorem applications

Proofs for various applications of Theorem 1 from the main text, restated below for convenience, are given in this supplementary note.

Theorem 1. Assume $d_c < d_b$ and $\mathcal{B}$ maps some pure state $[\psi]_a$ to an output $\mathcal{B}([\psi]_a)$ of rank d_c, then $\mathcal{Q}^{(1)}(\mathcal{B}) > 0$.

4.1 Channel with equal output and environment dimension

In the main text we argued how Theorem 1 applies to channels $\mathcal{B}$ with $d_c \geq d_b$. Here we give an explicit example where Theorem 1 applies when $d_c = d_b = 3$. Consider an isometry $K : \mathcal{H}_a \mapsto \mathcal{H}_b \otimes \mathcal{H}_c$ given by

$$\begin{aligned} K|0\rangle &= \sqrt{1-p}|00\rangle + \sqrt{p}|11\rangle, \\ K|1\rangle &= |21\rangle, \\ K|2\rangle &= |12\rangle, \end{aligned} \tag{15}$$

where $0 \leq p \leq 1$, $\{|0\rangle, |1\rangle, |2\rangle\}$ is the standard orthonormal basis. At $p = 0$ each channel in the $(\mathcal{B},\mathcal{C})$ pair defined by J is antidegradable and has zero quantum capacity. For all other values of p, Theorem 1 shows that both channels in the pair $(\mathcal{B},\mathcal{C})$ have positive $\mathcal{Q}^{(1)}$. We apply this theorem to sub-channels of $\mathcal{B}$ and $\mathcal{C}$.

First, consider $\mathcal{B}'$, a sub-channel of $\mathcal{B}$ obtained by restricting the input of $\mathcal{B}$ to a subspace spanned by $\{|0\rangle, |1\rangle\}$. This sub-channel $\mathcal{B}'$ satisfies the conditions of Theorem 1. At $p = 1$, $\mathcal{B}'$ has a two-dimensional output which is larger than its one dimensional environment, and $\mathcal{B}'$ maps a pure state input [1] to a one-dimensional output. For $0 < p < 1$, $\mathcal{B}'$ has an output dimension 3 which is larger than its environment dimension 2, and $\mathcal{B}'$ maps [0] to an output of rank 2.

Next, consider $\tilde{\mathcal{C}}$, a sub-channel of $\mathcal{C}$ obtained by restricting the input of $\mathcal{C}$ to a subspace spanned by $\{|0\rangle, |2\rangle\}$. This sub-channel $\mathcal{C}'$ also satisfies the conditions of Theorem 1. At $p = 1$, $\mathcal{C}'$ has a two-dimensional output which is larger than its one dimensional environment, and $\mathcal{C}'$ maps a pure state input [1] to a one-dimensional output. For $0 < p < 1$, $\mathcal{C}'$ has an output dimension 3 which is larger than its environment dimension 2, and $\mathcal{C}'$ maps [0] to an output of rank 2.

4.2 Incomplete Erasure Channels

In the main text we introduced the incomplete erasure channel $\mathcal{C}$. We claimed that any zero quantum capacity qubit channel $\mathcal{C}_1$ with a qubit environment can be used to assist this incomplete erasure channel in sending quantum information. We also asserted that our Theorem can be used to prove this claim. In what follows we provide a systematic treatment to support our statements. For completeness we introduce the generalized erasure channel pair [7] with superoperators,

$$\begin{aligned}\mathcal{B}(A) &= (1-\lambda)\mathcal{B}_1(A) \bigoplus \lambda\mathcal{T}(A), \quad \text{and} \\ \mathcal{C}(A) &= (1-\lambda)\mathcal{C}_1(A) \bigoplus \lambda\mathcal{I}(A),\end{aligned} \tag{16}$$

where $(\mathcal{B}_1, \mathcal{C}_1)$ is an arbitrary channel pair, $\mathcal{T}(A) = \mathrm{Tr}(A)[0]$ is the trace channel, $\mathcal{I}(A) = A$ is the identity channel, $\bigoplus$ is the direct sum symbol, and $0 \leq \lambda \leq 1$. Here $\mathcal{C}$ is the incomplete erasure channel. We are interested in the case where $(\mathcal{B}_1, \mathcal{C}_1)$ is some qubit channel pair such that $\mathcal{Q}(\mathcal{C}_1) = 0$. Up to local unitaries, any such channel pair is generated by an isometry $K_1 : \mathcal{H}_a \mapsto \mathcal{H}_{b1} \otimes \mathcal{H}_{c1}$ of the form [8,9]

$$\begin{aligned}K_1|0\rangle &= \sqrt{1-mp}\,|00\rangle + \sqrt{mp}\,|11\rangle, \\ K_1|1\rangle &= \sqrt{1-p}\,|10\rangle + \sqrt{p}\,|01\rangle,\end{aligned} \tag{17}$$

where $0 \leq m \leq 1$ and $0 \leq p \leq 1/2$ such that $\mathcal{C}_1$ is antidegradable and $\mathcal{Q}(\mathcal{C}_1) = 0$. This zero capacity qubit channel $\mathcal{C}_1$ has a qubit environment and a noise parameter p. The second channel parameter m can describe the type of noise, for instance at $m = 0$, $\mathcal{C}_1$ is an amplitude damping channel, and at $m = 1$ $\mathcal{C}_1$ is a measure-and-prepare channel [10].

At $p = 0$, $\mathcal{C}_1$ is the trace channel $\mathcal{T}$ and $\mathcal{C}$ is an erasure channel with erasure probability $1-\lambda$. For this erasure channel both $\mathcal{Q}^{(1)}(\mathcal{C})$ and $\mathcal{Q}(\mathcal{C})$ equal $\max(0, 2\lambda - 1)$ [11] i.e., they are both zero for all $0 \leq \lambda \leq 1/2$. But as soon as p is made positive by an arbitrarily small amount, $\mathcal{Q}^{(1)}(\mathcal{C})$ becomes positive over the entire $\lambda > 0$ range. This positivity comes from applying Theorem 1 to the $\mathcal{C}$ channel : $d_b = 3 < d_c = 4$ and a pure state $|\psi\rangle_a = (|0\rangle_a + i|1\rangle_a)/\sqrt{2}$ is mapped to an output $\mathcal{C}([\psi]_a)$ of rank d_b.

4.3 Corollaries

Next, we restate and prove Corollary 1 from the main text and show how it can be applied to the complement qubit channels.

Corollary 1. Suppose $d_a > 1$ and $d_b > d_a(d_c - 1)$ then $\mathcal{Q}^{(1)}(\mathcal{B}) > 0$.

Proof. Follows from Theorem 1 by noting that if $d_a > 1$ and $d_b > d_a(d_c - 1)$ then $d_c < d_b$ and there exists some pure state $[\psi]_a$ whose output, $\mathcal{B}([\psi]_a)$, has rank d_c. The existence of such a pure state $[\psi]_a$ can be shown by contradiction as follows. Given $d_b > d_a(d_c - 1)$ and $d_a > 1$, assume no pure state $[\psi]_a$ has an output $\mathcal{B}([\psi]_a)$ of rank d_c. As discussed in the previous section, any pure state $[\psi]_a$ has outputs $\mathcal{B}([\psi]_a)$ and $\mathcal{C}([\psi]_a)$ of equal rank. Consequently, this rank can never be greater than $\min(d_b, d_c) = d_c$, and by assumption this rank is not d_c, hence all pure states must be mapped by $\mathcal{B}$ to outputs of rank at most $d_c - 1$. Given any orthonormal basis $\{|\alpha_i\rangle_a\}$ of $\mathcal{H}_a$, any pure state $[\alpha_i]_a$ must get mapped by $\mathcal{B}$ to an output $\mathcal{B}([\alpha_i]_a)$ of rank at most $d_c - 1$. Consequently the sum of operators,

$$\sum_{i=1}^{d_a} \mathcal{B}([\alpha_i]_a) = \mathcal{B}\big(\sum_{i=1}^{d_a} [\alpha_i]_a\big) = \mathcal{B}(I_a), \tag{18}$$

evaluated using linearity of $\mathcal{B}$ and $I_a = \sum_i [\alpha_i]_a$, has rank at most $d_a(d_c - 1)$. By definition (see comments below (2)), $\mathcal{B}(I_a)$ has rank d_b, thus we arrive at an inequality $d_b \leq d_a(d_c - 1)$, which contradicts our starting condition $d_b > d_a(d_c - 1)$. Thus given that $d_b > d_a(d_c - 1)$ and $d_a > 1$ one cannot assume that no pure state $[\psi]_a$ has an output $\mathcal{B}([\psi]_a)$ of rank d_c i.e., there must be some pure state input $[\psi]_a$ with output $\mathcal{B}([\psi]_a)$ of rank d_c.

■

In the main text, we claimed that Corollary 1, stated above, shows that any qubit channel with a three of four dimensional environment has a complement with non-zero quantum capacity. Now, we prove this claim. Replacing $\mathcal{B}$ with its complement $\mathcal{C}$ in Corollary 1 results in the following statement: if $d_a > 1$ and $d_c > d_a(d_b - 1)$ then $\mathcal{Q}^{(1)}(\mathcal{C}) > 0$. When $d_a = d_b = 2$, i.e., $\mathcal{B}$ is a qubit channel, then the above statement leads to

Corollary 2. The complement $\mathcal{C}$ of a qubit channel $\mathcal{B}$ has strictly positive $\mathcal{Q}^{(1)}(\mathcal{C})$ whenever $d_c > 2$.

Supplementary Note 5: Qutrit Channel and Coherent information

We discuss properties of the isometry $J_1 : \mathcal{H}_{a1} \mapsto \mathcal{H}_{b1} \otimes \mathcal{H}_{c1}$ in eq. (6) of the main text. Below that equation, we stated that an exchange of s with $1-s$ can be achieved by local unitaries. These unitaries exchange $|1\rangle$ and $|2\rangle$ in $\mathcal{H}_{a1}$, and exchange $|0\rangle$ and $|1\rangle$ in both $\mathcal{H}_{b1}$ and $\mathcal{H}_{c1}$. The isometry J_1 gives rise to the channel pair $(\mathcal{B}_1, \mathcal{C}_1)$. For the full range of $0 \leq s \leq 1/2$ values, $\mathcal{Q}^{(1)}(\mathcal{B}_1)$ is strictly positive and is simply given by $\Delta(\mathcal{B}_1, \rho^*_{a1})$ where,

$$\rho^*_{a1} = (1-w)[0]_{a1} + w[1]_{a1}, \tag{19}$$

and $0 < w < 1$. A proof of this previous statement is discussed below. For the purposes of this discussion, we can get away with a more concise notation than the one in the rest of this supplementary information write-up: let $\rho \in \hat{\mathcal{H}}_{a1}$ be any density operator with matrix elements $\rho^{ij} = \langle i|\rho|j\rangle$ where $i,j \in \{0,1,2\}$, S_b and S_c be the von-Neumann entropies of $\mathcal{B}_1(\rho)$ and $\mathcal{C}_1(\rho)$, respectively, and $\Delta := S_b - S_c$ be the entropy bias whose maximum over ρ gives the channel coherent information $\mathcal{Q}^{(1)}(\mathcal{B}_1)$.

For any input density operator ρ setting $\rho^{01} = \rho^{02} = 0$ increases S_b without changing S_c, so we can always maximize Δ using a density operator of the form,

$$\rho = \begin{pmatrix} 1-w & 0 & 0 \\ 0 & w(1+z)/2 & w(x+iy)/2 \\ 0 & w(x+iy)/2 & w(1-z)/2 \end{pmatrix}, \tag{20}$$

written in the standard basis using real parameters $0 \leq w \leq 1$ and x, y, and z all between -1 and 1 such that $x^2 + y^2 + z^2 \leq 1$. In this parametrization, S_b is independent of x, y, and z, on the other hand S_c depends on x, y through $x^2 + y^2$ so we set $y = 0$. Next, at $y = 0$, S_c in concave in $x^2 + z^2$ thus the maximum value of Δ occurs when S_c is minimum at $x^2 + z^2 = 1$. These simplifications leave two free parameters $0 \leq w \leq 1$ and $-1 \leq z \leq 1$. For a fixed w, at $s = 1/2$ the entropy S_c is independent of z, and for other $0 \leq s < 1/2$, S_c is monotone decreasing in z, as a consequence for all $0 \leq s \leq 1/2$, we set $z = 1$ to maximize Δ at

$$\rho = (1-w)[0] + w[1]. \tag{21}$$

For any $0 \leq s \leq 1/2$, if we let w in (21) be a small positive number ϵ, then the entropy S_b has an $\epsilon \log$-singularity, while S_c doesn't, as a consequence $\mathcal{Q}^{(1)}(\mathcal{B}_1) > 0$. Since $\mathcal{Q}^{(1)}(\mathcal{B}_1)$ is positive, its value can be obtained my maximizing Δ over ρ in (21) by varying w between zero and one while excluding $w = 0$ and $w = 1$ where $\Delta = 0$.

References

[1] Mark M. Wilde. *Quantum Information Theory*. Cambridge University Press, 2 edition, 2017. doi:10.1017/9781316809976.

[2] I. Devetak and P. W. Shor. The capacity of a quantum channel for simultaneous transmission of classical and quantum information. *Communications in Mathematical Physics*, 256(2):287–303, 2005. doi:10.1007/s00220-005-1317-6.

[3] M. Fannes. A continuity property of the entropy density for spin lattice systems. *Communications in Mathematical Physics*, 31(4):291–294, Dec 1973. doi:10.1007/BF01646490.

[4] Koenraad M R Audenaert. A sharp continuity estimate for the von neumann entropy. *Journal of Physics A: Mathematical and Theoretical*, 40(28):8127–8136, Jun 2007. doi:10.1088/1751-8113/40/28/s18.

[5] R Alicki and M Fannes. Continuity of quantum conditional information. *Journal of Physics A: Mathematical and General*, 37(5):L55–L57, Jan 2004. doi:10.1088/0305-4470/37/5/l01.

[6] Andreas Winter. Tight uniform continuity bounds for quantum entropies: Conditional entropy, relative entropy distance and energy constraints. *Communications in Mathematical Physics*, 347(1):291–313, Oct 2016. doi:10.1007/s00220-016-2609-8.

[7] Vikesh Siddhu and Robert B. Griffiths. Positivity and nonadditivity of quantum capacities using generalized erasure channels, Mar 2020. arXiv:2003.00583.

[8] Michael M. Wolf and David Pérez-García. Quantum capacities of channels with small environment. *Phys. Rev. A*, 75:012303, Jan 2007. doi:10.1103/PhysRevA.75.012303.

[9] Mary Beth Ruskai, Stanislaw Szarek, and Elisabeth Werner. An analysis of completely-positive trace-preserving maps on m2. *Linear Algebra and its Applications*, 347(1–3):159 – 187, 2002. doi:10.1016/S0024-3795(01)00547-X.

[10] Michael Horodecki, Peter W. Shor, and Mary Beth Ruskai. Entanglement breaking channels. *Reviews in Mathematical Physics*, 15(06):629–641, 2003. doi:10.1142/S0129055X03001709.

[11] Charles H. Bennett, David P. DiVincenzo, and John A. Smolin. Capacities of quantum erasure channels. *Phys. Rev. Lett.*, 78:3217–3220, Apr 1997. doi:10.1103/PhysRevLett.78.3217.
